# Indocyanine Green Video Angiography Predicts Outcome of Extravasation Injuries

**DOI:** 10.1371/journal.pone.0103649

**Published:** 2014-08-21

**Authors:** Werner Haslik, Ursula Pluschnig, Günther G. Steger, Christoph C. Zielinski, K. F. Schrögendorfer, Jakob Nedomansky, Rupert Bartsch, Robert M. Mader

**Affiliations:** 1 Division of Plastic and Reconstructive Surgery, Department of Surgery, Comprehensive Cancer Center of the Medical University of Vienna, Vienna, Austria; 2 Department of Medicine I, Clinical Division of Oncology, Comprehensive Cancer Center of the Medical University of Vienna, Vienna, Austria; Medical University of Graz, Austria

## Abstract

**Background:**

Extravasation of cytotoxic drugs is a serious complication of systemic cancer treatment. Still, a reliable method for early assessment of tissue damage and outcome prediction is missing. Here, we demonstrate that the evaluation of blood flow by indocyanine green (ICG) angiography in the extravasation area predicts for the need of surgical intervention.

**Methods:**

Twenty-nine patients were evaluated by ICG angiography after extravasation of vesicant or highly irritant cytotoxic drugs administered by peripheral i.v. infusion. Tissue perfusion as assessed by this standardized method was correlated with clinical outcome.

**Results:**

The perfusion index at the site of extravasation differed significantly between patients with reversible tissue damage and thus healing under conservative management (N = 22) versus those who needed surgical intervention due to the development of necrosis (N = 7; *P* = 0.0001). Furthermore, in patients benefiting from conservative management, the perfusion index was significantly higher in the central extravasation area denoting hyperemia, when compared with the peripheral area (*P* = 0.0001).

**Conclusions:**

In this patient cohort, ICG angiography as indicator of local perfusion within the extravasation area was of prognostic value for tissue damage. ICG angiography could thus be used for the early identification of patients at risk for irreversible tissue damage after extravasation of cytotoxic drugs.

## Introduction

Extravasation as a complication of chemotherapy administration is defined as the unintended instillation or leakage of cytotoxic agents into the perivascular space or subcutaneous tissue. The incidence of extravasations ranges from 0.45% up to 6.4%, with higher figures observed in patients who had previously received multiple lines of chemotherapy [1], [2]. The degree of local damage depends upon the toxic potential of the extravasated compound and the amount of drug delivered to the perivascular tissue [3].

Usually, three different classes of cytotoxic compounds are distinguished: i) non-vesicant substances which do not cause local irritations in case of extravasation; ii) irritant substances which cause pain, tissue irritation and swelling, but do not lead to tissue necrosis; iii) vesicant substances, which can cause ulcerations and necrosis necessitating surgical débridement and reconstruction with skin grafts, local flaps or even free flap coverage [4].

As the management of extravasations of cytotoxic drugs is mostly based upon retrospective evaluations of case reports and some data from animal models, the level of evidence of recommendations for clinical management of extravasation events is low. In addition, methods to objectively document the extent of tissue damage are based on photographic documentation alone. This technique is limited insofar, as sequelae including demarcation of skin, ulceration and necrosis may present weeks after the extravasation event. Typically, necrosis is detected 3–4 weeks after the extravasation event. Moreover, superficial alterations of the skin may not represent the true extent of subcutaneous tissue damage. Therefore, novel methods for the reliable identification of the extent of local tissue damage as well as early predictors of outcome are urgently warranted.

As extravasations are frequently associated with injury related venous thrombosis, one possibility to illustrate the extent of the affected extravasation area is to assess skin blood perfusion, which may be done by indocyanine green (ICG) angiography. This procedure is well-established in ophthalmology, burn and trauma management as well as surgical reconstruction for the assessment of tissue perfusion. The basic principle of all these applications is to enable an objective measurement of perfusion and to detect perfusion anomalies, which will eventually lead to necrosis. Therefore, ICG angiography has a predictive value, which is not available by clinical assessment only. In trauma management, ICG is able to predict occurrence of necrosis leading to a better management of the patients. In burn patients, ICG angiography was used for objectively estimating burn wound depth thereby assisting in the rational assessment of treatment options [5], [6], [7]. In oncology, the use of ICG angiography is so far limited to the detection of sentinel lymph nodes [8].

In contrast to non-vesicant and irritant cytotoxics, vesicants are prone to generate irreversible tissue damage at the extravasation site. Thus, extravasated vesicant cytotoxics define a subgroup of patients at high risk and in urgent need of an objective procedure to reliably assess extent and progress of local damage. In our department, decision-making for the treatment of extravasation injuries was based only on clinical assessment in the past. The aim of this study was to evaluate the impact of ICG angiography on determination of the extent of extravasation injuries and to assess its prognostic role with regards to clinical outcome.

## Materials and Methods

### Patients

Twenty-nine patients with extravasation of cytotoxics (vesicant or highly irritant agents) administered by peripheral i.v. infusion were included in this study. The study population included 22 female and seven male subjects (median age: 64 years, range: 33–81 years).

Inclusion criteria for the evaluation by ICG angiography were:

1. Patients with a substantial amount of extravasated compound (when a substantial part of the infusion volume was suspected to be administered in the subcutaneous space at that time), where the possibility of necrosis was highly suspected;
2. Patients with ongoing and persistent objective symptoms one or two weeks after the extravasation event or patients suffering from non-healing injuries.

The study was approved by the Institutional Review Board of the University Hospital Vienna. All patients gave written informed consent according to the institutional guidelines. Application of ICG for angiographic purposes was performed in accordance with the ethical regulations of the Medical University of Vienna.

### ICG angiography

ICG angiography uses the tricarbocyanine dye ICG that binds quantitatively to plasma proteins. Due to this very rapid binding to plasma proteins after intravenous injection, there is no distribution into the interstitial space and ICG remains located exclusively in the intravascular space thereby allowing for the monitoring of superficial blood perfusion. With a plasma half-life of three to four min, ICG is metabolised by the liver and excreted into the bile. As described by Giunta and co-workers, the absorption and emission peaks in the near infrared spectral range (805 nm and 835 nm) lie within the optical window of the skin, enabling a penetration depth of at least three mm [9].

A single dose of 0.2 mg/kg ICG (ICG-Pulsion, Pulsion Medical Systems, Munich, Germany) was administered by intravenous bolus injection. The perfusion of the affected area was measured using dynamic laser-fluorescence-videography (IC-VIEW, Pulsion Medical Systems) [5], [6]. This system contains a digital video camcorder combined with a near infrared light source. The results were recorded digitally allowing for real time viewing as well as quantitative evaluation performed by quantification software (IC-CALC, Pulsion Medical Systems). Different regions of interest (ROI) were defined: ROI 1 was centered in the middle of the extravasation lesion (red colour). ROI 2 was defined in the surrounding margin, but within the affected area of the lesion (blue colour). The fluorescence intensity of normal, well-perfused tissue (unaffected skin) was used for reference (green colour). Moreover, an ICG reference card (supplied by the manufacturer) was included in each measurement and used to normalize the recorded fluorescence data (yellow colour). Perfusion index and maximum pixel intensity of the defined ROI were calculated by IC-CALC software in each patient.

ICG fluorescence intensity throughout the measurement is drawn as a curve with affiliated slopes during signal increase. The software calculates the perfusion index in these regions as steepness of the curve of fluorescence intensity over time (pixel intensity per second) in comparison to the reference area (results in percent). The perfusion index is a parameter that gives more valid information on the quality of perfusion than the maximum ICG-fluorescence intensity alone. This parameter represents the information on the speed of filling with ICG, which correlates with the extent of blood supply [9]. The median period of time after the extravasation event during which patients were monitored for “conservative management” or “surgical intervention” outcomes was 44 days (range: 14–240 d).

### Statistics

Outcome parameters obtained after ICG angiography, i.e. perfusion index and maximum pixel intensity, were compared by the Mann-Whitney test (patients with/without surgical intervention). According to the dependence of outcome variables, paired or non-paired statistical comparisons were calculated using GraphPad Prism version 4 software.

## Results

In this exploratory study, 29 patients (female: 22; male: 7) with an extravasation of cytotoxic agents after i.v. administration were included. In 16 subjects, the extravasation was noted during infusion or on the same day, while in the reminders, the event was reported on the days thereafter. The ICG angiography took place within a median time-span of 12 days after the extravasation event. The extravasated compounds were mostly anthracyclines (epirubicin: 12, doxorubicin: 4, mitoxantrone: 1); other cases were caused by extravasations with vinorelbine (*N* = 5), platinum compounds (*N* = 3), trabectedin (*N* = 2), docetaxel (*N* = 1), and one not specified case (extravasation occurring during CHOP polychemotherapy). As all cytotoxics had the potential to cause severe tissue damage, there was no particular cytotoxic agent that was associated with more severe necrosis. The extravasation site was forearm (*N* = 14), dorsum of hand (*N* = 9), upper arm (*N* = 3), and antecubital fossa (*N* = 3).

**Figure 1:**
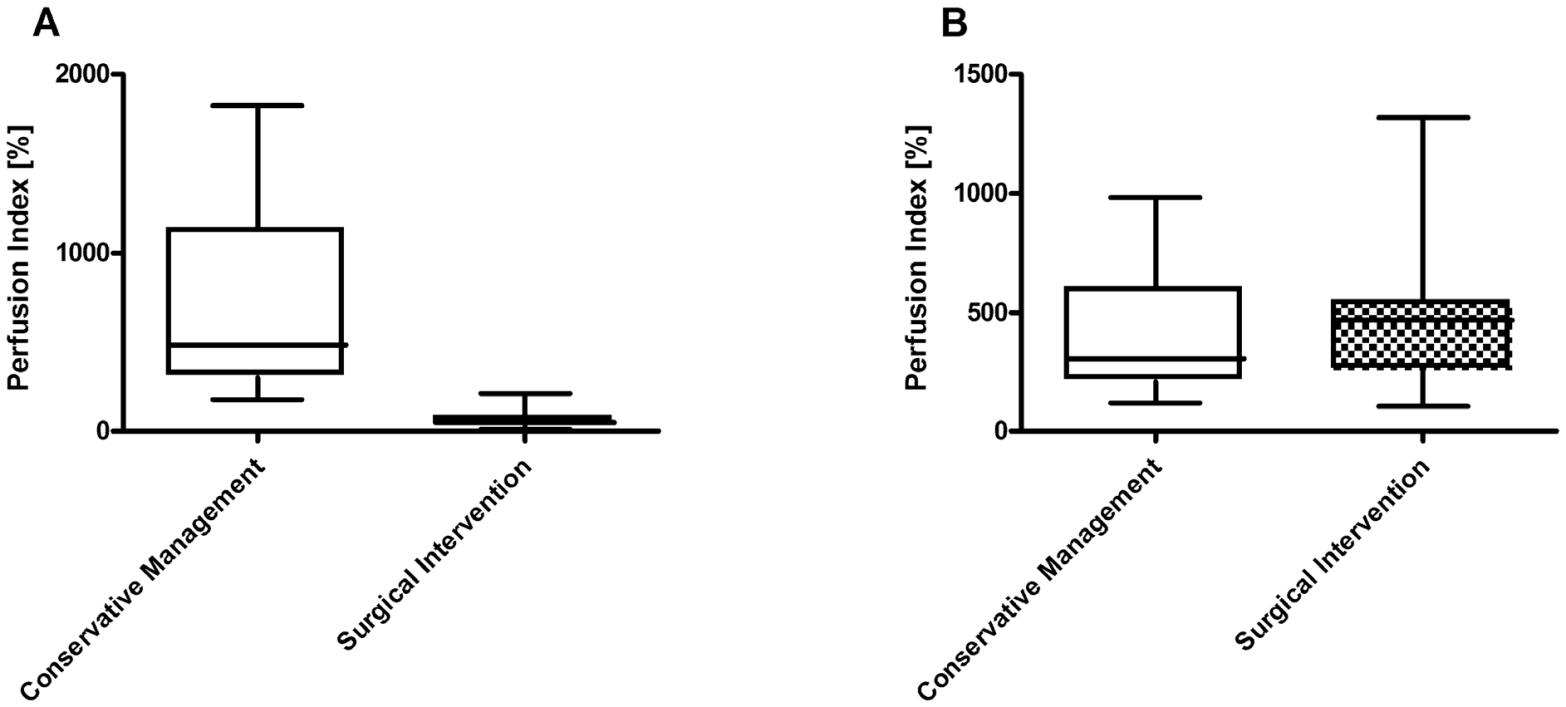
Perfusion at central area (A) and peripheral area (B) of the extravasation lesion. Box plots represent cutaneous blood flow as assessed by the perfusion index in patients with conservative clinical management (*N* = 22) versus those requiring subsequently surgical intervention (*N* = 7); *statistical significance p<0.05.

Considering the perfusion index at extravasation areas, there was a highly significant difference between patients with conservative management (*N* = 22) and patient who needed surgical intervention (*N* = 7) due to irreversible tissue damage resulting in necrosis (*P* = 0.0001). This difference was restricted to the central area of the extravasation lesion at the injection site (Figure 1A) and was not observed in peripheral areas of the lesion (Figure 1B). Similarly, there was a highly significant difference between patients with conservative management and patients undergoing surgical intervention when considering another monitoring parameter, the maximum pixel intensity in the central area (*P* = 0.0012).

**Figure 2:**
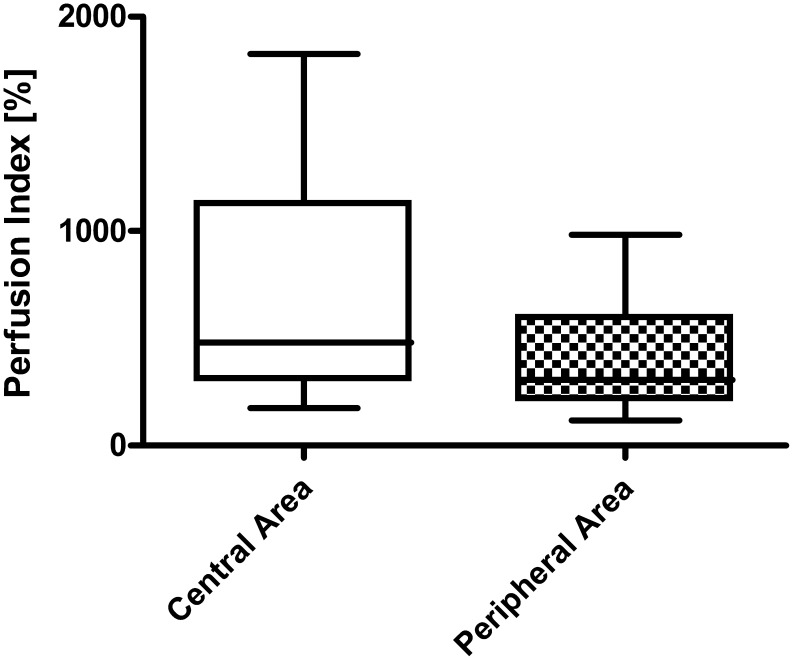
Perfusion in patients not requiring surgery: central area versus peripheral area of the extravasation lesion. Box plots represent cutaneous blood flow as assessed by the perfusion index in patients relying on conservative clinical management (*N* = 22).

**Figure 3:**
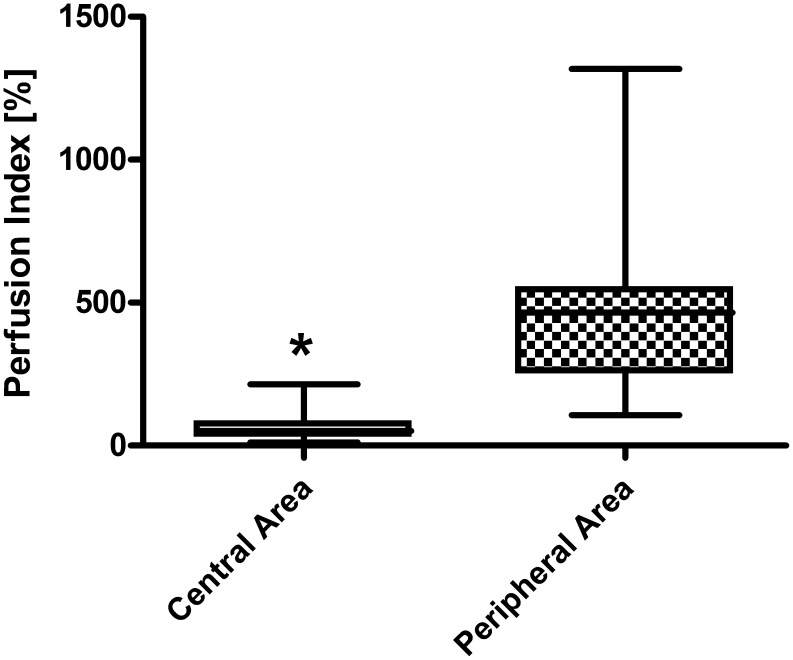
Perfusion in patients requiring surgery: central area versus peripheral area of the extravasation lesion. Box plots represent cutaneous blood flow as assessed by the perfusion index in patients requiring surgical intervention after extravasation of cytotoxics (*N* = 7); *statistical significance p<0.05.

With regard to patients not requiring surgical interventions and thus healing under conservative management, the perfusion index was significantly higher in the central area of the lesion when compared with the peripheral area of the lesion indicative for a hyperaemic situation in the centre (*P* = 0.0001; Figure 2). In contrast, patients later requiring surgical intervention always showed a diminished perfusion index in the central area of the lesion when compared with the peripheral area (*P* = 0.0156) indicating a massive hypoperfusion as early indicator of irreversible tissue damage (Figure 3).

With a perfusion index set at a threshold level of 100%, a single patient undergoing later surgery presented with a higher perfusion index (sensitivity: 86%). Remarkably, there were no false positive patients as all patients with successful conservative management had perfusion indices higher than 100% in the central area of the lesion as well as in the peripheral area.

As we wanted to use the method as early predictive tool, we used only the first ICG evaluation for outcome reporting. In selected patients (n = 8), repeated measurements could be performed. In two of them, hypoperfusion in the central area of the lesion persisted throughout the observation period resulting eventually in necrosis and surgical intervention (range of the perfusion index: 36%–93%). In contrast, patients with conservative management, the perfusion index at the site of extravasation (central area) always considerably exceeded the threshold level of 100% (range of the perfusion index: 200%–1440%).

## Discussion

This investigation was conducted in order to assess potential complications of chemotherapy extravasation by early prediction of sequelae, particularly irreversible tissue damage resulting in necrosis that will ultimately require surgical intervention and render conservative treatment futile. Tissue necrosis is a characteristic of vesicant cytotoxics, but has also sporadically been observed with compounds classified as irritant and even the less harmful group of non-vesicant agents. Therefore, the central issue to be addressed after extravasation is the early prediction of the necessity of surgical intervention as this knowledge determines intensity of follow-up visits, planning of surgery, and finally continuation of chemotherapy cycles with a minimum of delay. The exact extent of the extravasation area as well as the identification of irreversible tissue damage cannot be defined by macroscopic methods. Indeed, irreversible breakdown of subcutaneous tissue architecture may occur unnoticed well below the seemingly intact epidermal layer. In this region, spontaneous healing may no longer be expected.

Frequently, extravasation of vesicant agents is associated with thrombotic events thus further reducing local clearance of extravasated agents. In this context, thrombosis prevention with heparin has proven efficacious in an animal model by reducing extent and frequency of doxorubicin-related ulcerations, but was of limited effect in humans [10], [11]. Enhanced tissue residence time of vesicants due to thrombosis in combination with higher subcutaneous concentrations of toxic compounds is therefore expected to pose a high risk for subsequent progression to tissue necrosis. We therefore hypothesised that early diagnosis of hypoperfusion of areas affected by extravasation would contribute to the prediction of an unfavourable clinical course. ICG angiography is a non-invasive technique to quantify skin perfusion down to a depth of approximately three millimetres. Upon several occasions, ICG angiography has proven to be a useful tool, which can be readily applied after burns and flap transplantation to discriminate perfused from non-perfused tissue areas. Other indications are assessment of liver function and cardiac output as well as ophthalmic examinations. This qualifies ICG angiography as a well-established and safe technique. As recording of blood flow is usually concluded within five minutes, the overall analysis time needed is approximately 30 minutes. As ICG angiography examines the upper layer of the skin, all dressings need to be removed and all measures, which may influence blood flow in the affected area are temporarily suspended including antidote administration [12]. In our experience, this is compatible with the current protocols of antidote administration (topical cooling, topical heat, application of DMSO or s.c. injection of hyaluronidase). The only contraindication against the use of ICG known so far is allergy against iodine.

In this study, we provide information about results of ICG angiography in two cohorts of patients after extravasation with vesicant cytotoxics. Of note, none of the patients with a perfusion index above 100% in the central area of the affected lesion developed subsequent tissue necrosis. This practically relevant information might be useful for further planning of the patient’s follow-up visit and - if confirmed in a larger prospective series - may prove beneficial for all persons experiencing an extravasation. All other subjects not fulfilling this criterion may be considered at risk for developing severe sequelae such as necrosis and ultimately surgical intervention thus requiring a much closer attention and a more intense follow-up schedule.

As outlined, this observation may be explained by the fact that extravasations, particularly those of vesicants, are frequently associated with thrombosis in the affected tissue.

In contrast, a hyperaemic situation indicated an intact architecture of skin and blood vessels able to detoxify the extravasation area. This observation has been corroborated by histopathological examinations in our group (data not shown). This distinction cannot be made by macroscopic examination alone, which may even be misleading. Particularly in early stages of developing skin toxicity, a reasonable prediction of the imminent tissue damage based upon a purely macroscopic assessment is almost impossible even by experienced oncologic surgeons, but is feasible after ICG angiography. Despite these advantages, ICG angiography is limited in its predictive value to extravasations associated with local thrombotic events, which are frequently but not always observed. Due to the penetration depth of approximately three millimetres, deeper thrombosis and tissues obstruction in layers below the subcutaneous strata are likely to yield false negative results. Other issues to be elucidated in further investigations are the selection of patients, the predictive role in other compounds besides the investigated vesicants, the exact planning of ICG angiography and possible repetitions of ICG-based evaluations. A fine tuning of the cut-off values for discrimination may also be necessary, although the perfusion index above 100% may be considered a reasonable starting point for the follow-up management. As blood perfusion may also be examined by other techniques, non-invasive procedures such as the laser Doppler technique of skin blood measurement may pose an attractive alternative method [13,14]. This imaging technique is not dependent on the i.v. injection of a dye solution and may be performed in the absence of darkroom conditions.

Study limitations are that the two observers who were not independent from the treating team performed the ICG angiography and that ICG analyses were not performed in a blinded fashion. The relatively small number of 29 patients and the heterogeneity of the population can be considered another study limitation. However, given the fact that extravasation with a considerable amount of vesicant cytotoxics is a rare event and considering the highly significant difference between both treatment groups, 29 patients appear to be sufficient to postulate the value of ICG angiography in predicting the course of the extravasation event.

In conclusion, these data show for the first time that days after an extravasation event, a perfusion index <100% in the central area of the lesion is a strong indicator for patients later requiring surgical intervention. Likewise, a perfusion index >100% strongly predicts spontaneous healing under conservative management. The results of this prospective trial therefore indicate that ICG angiography objectively documents skin perfusion and helps defining the extent of injury after extravasation of cytotoxics. The early understanding of prognosis of subcutaneous skin injury may be very useful information to guide the decision for conservative or surgical intervention, the intensity of follow-up visits, and further planning of chemotherapy.
